# Modular design of curved beam-based recyclable architected materials

**DOI:** 10.1016/j.heliyon.2023.e21557

**Published:** 2023-11-07

**Authors:** Hongyi Yao, Xiaoyu Zhao, Shengli Mi

**Affiliations:** a Bio-manufacturing Engineering Laboratory, Tsinghua Shenzhen International Graduate School, Tsinghua University, Shenzhen, China; b Department of Mechanical Engineering, Tsinghua University, Beijing, China

**Keywords:** Architected materials, Initially curved beam, Modular design of materials, Complex geometrical reconstruction, Sustainable recycling

## Abstract

Advances in manufacturing technologies have enabled architected materials with unprecedented properties. These materials are typically irreversibly designed and fabricated with characteristic geometries and specific mechanical properties, thus rendering them suitable for pre-specified requests. However, these materials cannot be recycled or reconstructed into different shapes and functionalities to economically adapt to various environments. Hence, we present a modular design strategy to create a category of recyclable architected materials comprising elastic initially curved beams and rigid cylindrical magnets. Based on numerical analyses and physical prototypes, we introduce an arc-serpentine curved beam (ASCB) and systematically investigate its mechanical properties. Subsequently, we develop two sets of hierarchical modules for the ASCB, thus expanding the constructable shape of architected materials from regular cuboids to complex curved surfaces. Furthermore, we demonstrate that the magnets attached to the centers of specific serpentine patterns of the modules allows the effective in-situ recycling of the designed materials, including sheet materials for non-damage storage, bulk materials for tunable stiffness, and protective package boxes for reshaping into decorative lampshades. We expect our approach to improve the flexibility of architected materials for multifunctional implementation in resource-limited scenarios.

## 1 Introduction

The unique properties of architected materials (metamaterials), which are analogous to those of conventional materials, are determined by their unit cell structures and spatial arrangements. The mechanism of beam-based structured materials [[1], [2], [3], [4], [5]], the implementation of origami [[6], [7], [8], [9], [10], [11]] and kirigami [[12], [13], [14], [15], [16], [17]] structures, and the involvement of multiphysics interactions [[18], [19], [20], [21]] enrich the unusual mechanical characteristics of architected materials, including their shape-morphing capabilities [1,6,9,10,22,23], compliance characteristics [[12], [13], [14]], programmable properties [9,11,17,19], and topologies [18,[24], [25], [26], [27]]. However, most architected materials, such as industrial recyclable materials including paper, plastic, and glass, are manufactured for specific purposes; furthermore, their shapes and mechanical properties cannot be altered easily.

The in-situ recycling of materials and equipment is crucial for achieving sustainable living under limited resources, e.g. living in uninhabited deserts, enclosure rooms inside space stations, and oceangoing ships and submarines with no sustainable supplies. Reusable materials such as paperboard or plastic bags typically require additional tools and adhesives to achieve reconfigured shapes or functions. Unless expertise is applied, these processes will be time consuming and irreversible. Moreover, recycling the constituent components of useless materials typically requires specific chemical processing and large-scale production lines that are not suitable for the extreme conditions above. Hence, materials that can realise rapid, reversible, and efficient recycling at no additional cost must be developed urgently. Modular materials with tunable mechanical properties can potentially satisfy these requirements.

Significant efforts toward the design and fabrication of modular metamaterials [2,10,[28], [29], [30]] have shown that modules can be fabricated individually and assembled as complex hierarchical objects. Most origami-and kirigami-based modules are fully or partially created manually [9,10,29]. Beam-based modules possess complicated junctions, which result in unexpected stress concentrations and cannot be reconnected [2]. Moreover, the shapes of most modular architected materials are regular with zero curvature [2,9,10,30], which limits their application and necessitates further investigations into curved modular metamaterials. These factors hinder the efficient recycling of architected materials into various shapes with tunable properties and functions.

In this study, we address the abovementioned problems and develop a category of recyclable architected materials (RAMs). First, we report a universal curved beam-based building element that isolates concentrated stresses away from dominant deformation segments. Subsequently, we design two-dimensional (2D) and three-dimensional (3D) modules using the building elements and derive their variants with various mechanical properties using a heuristic algorithm. Next, we propose a magnetic stacking mechanism to create simple and reversible junctions between connected modules. By combining 2D and 3D modules with various topologies, complex shapes with negative, zero, and positive Gaussian curvature are achievable. Finally, we present three cases to reveal the potential applications of RAMs: 1) Storing a flat and soft sheet-like material with minimal space utilisation and in a damage-free manner; 2) adjusting the effective stiffness of a bulk-like material by filling elastic balls after recycling; 3) recycling a rectangular package, which serves as protection, into a stack of raw materials and assemble some of them into a convex surface to serve as lighting-area control.

## 2 Materials and method

### 2.1 Theoretical model and finite element analysis (FEA)

The details of the mathematical model for an arc-serpentine curved beam (ASCB) and its moduli are summarised in Sections 1–5 of the Supplementary Information. The optimisation method for designing the variants is described in Section 6 of the Supplementary Information. We implemented the models and algorithms in MATLAB (MathWorks, U.S.) and output them as STL files for fabrication. Subsequently, we conducted an FEA of the mechanical behaviours of the ASCB, which included M_0_ and M_3D_ series, using COMSOL Multiphysics (COMSOL Inc., Sweden). The Young's modulus and Poisson's ratio were set to 800 MPa and 0.33, respectively. For ASCB-6R (length: 120 mm), we fixed the left cross-section and applied a displacement (15 mm) or rotation (45°) boundary condition to the right cross-section. For M_0_ (length: 120 mm × 120 mm), an *anti*-periodic displacement (10 mm) boundary condition was applied to the ends of the x (or x and y) axial pair of ASCB-3Rs. For M_3D_ (length: 120 mm × 120 mm × 120 mm), we applied an *anti*-periodic displacement (10 mm) or rotation (45°) boundary condition on the specific areas of the quadrilateral connected M_0_s, where the magnets were affixed. We partitioned all geometries with tetrahedral elements and adopted a stationary solver with a fully coupled algorithm to calculate the solution.

### 2.2 Fabrication of modules

We fabricated 2D and 3D series modules using LCD 3D printing techniques (STERK4K; China). We shifted the supports of the printed models manually, painted them blue, and colour coded them based on their handedness. The cylindrical magnet used measured φ2mm×1mm. We adhered the magnet to the colored models using a one-component UV-light curving adhesive (Ergo 8500, Switzerland). The magnet costs were 16, 24, 32, 72, and 24 for M_2D_-I, M_2D_-II, M_2D_-III, M_2D_-IV, M_3D_-I, and M_3D_-II, respectively. Notably, the direction of the magnetic force was perpendicular to the attached surfaces, thus indicating that the strength of the magnetic attraction against shearing was much smaller than that against stretching. Hence, we uniformly distributed four docking points around the geometric centre on one M_0_ unit, thus preventing two stacked M_0_ units from relative translation under shearing. Meanwhile, shearing two stacked modules is an effective method for separating them.

### 2.3 Experimental test

The mechanical response of the seven variants and materials to uniaxial loading was investigated using a WD-10 compression testing machine. Each sample was compressed through the manual rotating handle of the machines until the loading number on the machine reached 8 N, which was the upper safe load limit. The entire process was captured in a video. Subsequently, we selected the number of loads and displacements at each second. The final data figures were plotted using the Origin Educational Edition (Origin Lab, U.S.). Furthermore, the differences and slopes for each sample point were calculated. Finally, we calculated the effective stiffness at the soft stage and the threshold strain using linear regression in Excel (Microsoft, U.S.).

## 3 Results

### 3.1 Design and characterization of ASCB

Beam-based unit cells (modules) of metamaterials are amenable to various fabrication procedures, ranging from subtractive to additive manufacturing. We used the classic beam model to design the modules of the recyclable metamaterials. In general, the geometries around the nodes and junctions of conventional beam-based unit cells must be augmented to strengthen their stiffness and avoid mechanical failure. This increases the undesirable consequences of node effects and causes researchers to compromise between stability and performance.

**Fig. 1:**
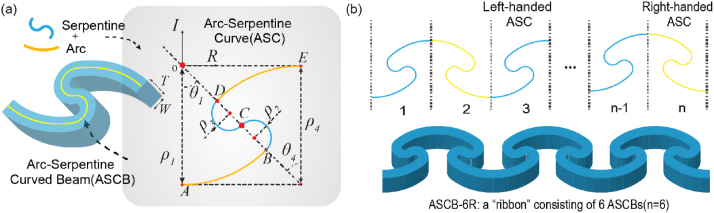
Design principles of arc-serpentine curved beam (ASCB) and the ribbon-shape ASCB-nR. (a) Geometric parameters of ASCB. (b) ASCB-6R consisting of six ASCBs with an antiferromagnetic order.

Herein, we propose a specific curved beam model as the building element. The neutral axis of this beam is a smooth curve comprising arcs and serpentine segments, which is referred to as an arc-serpentine curve (ASC) herein. Hence, we refer to our beam model as an ASCB. Based on the parameters defined in Fig. 1a, we developed a numerical model of the ASC (presented in the Supplementary Materials) to conduct a subsequent design process. We defined an ASC whose curvature transitioned from positive to negative as a left-handed ASC, and vice versa (Fig. 1). From a design perspective, we regarded one ASC as a ‘point’. Based on the generation of a line from connected points, we merged n ASCs into a line whose length is n times longer than that of a single ASC. The handedness along the line was configured in a staggered manner; thus, each pair of adjacent ASCs were opposite to each other (Fig. 1b). Finally, we set the cross-section of the ASCB as a rectangle.

**Fig. 2:**
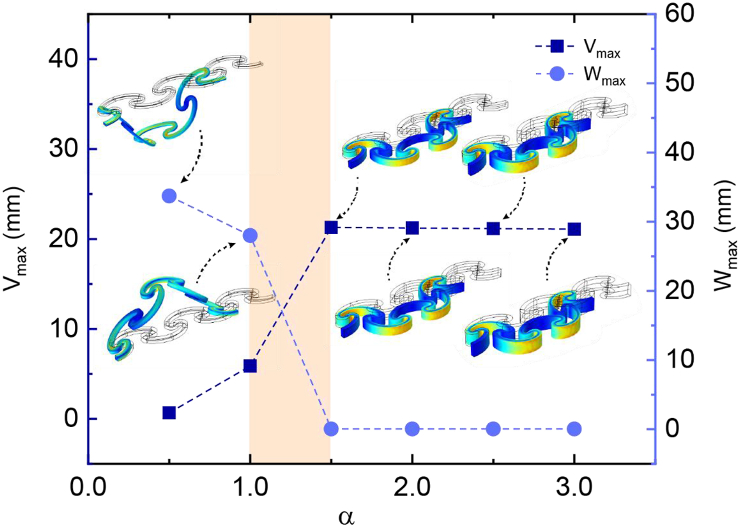
The response of ASCB-6R under uniaxial compression. The ratio of thickness to width (α) is ranged from 0.5 to 3.0.

First, based on FEA, we determined the appropriate ratio α of the thickness to the width of the cross-section to inhibit out-of-plane unstable elastic deformation (post-buckling behaviours) of ASCB-6R under uniaxial compression. The results showed that a critical α existed, at which the post-buckling direction of the ASCB-6R transitioned from out-of-plane to in-plane within the range of 1–1.5 (Fig. 2). Moreover, we exploited this in-plane buckling to prevent interference between the adjacent side surfaces of the two stacked ribbons. Subsequently, we set α = 2.

**Fig. 3:**
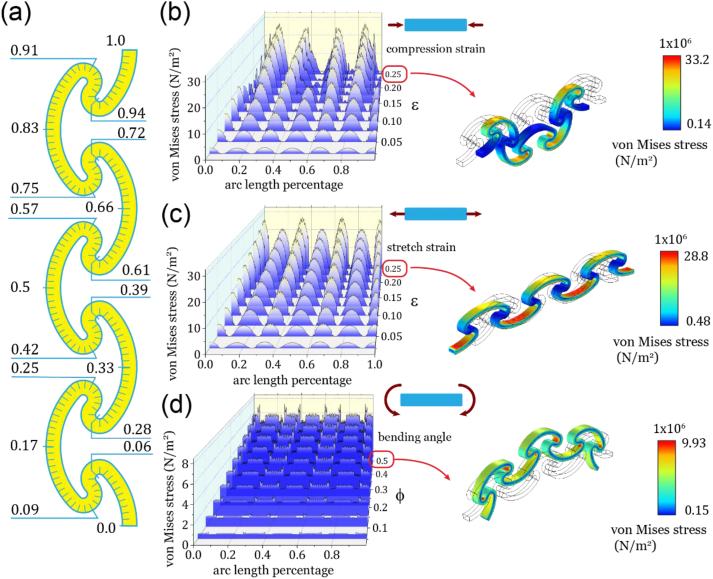
The finite element analysis (FEA) of ASCB-6R under different loading. (a) The demonstration of arc-length percentage of ASCB-6R. (b–d) From left to right, von Mises stress distribution along the arc-length percentage and elastic deformation of the sample at the maximum strain. From top to bottom, the loading situations are uniaxial compression, stretching and pure bending.

We investigated the mechanical behaviour of ASCB-6R under uniaxial compression, stretching, and bending via FEA (Fig. 3). The ‘ribbon’ exhibited a uniform and linear elongation and concave/convex deformation via stretching and bending. Meanwhile, the ASCB-6R subjected to compression showed a linear compressed deformation initially but became unstable and buckled in-plane when the strain exceeded 0.15. The results of these cases showed that the deformations occurred because of the bending of all the arc sections. This was due to the geometric configuration, in which the radii of curvature of the arc sections were much larger than those of the serpentine sections.

More importantly, the distribution of the von Mises stress along ASCB-6R (Fig. 3b–d) showed that the central areas of each serpentine segment exhibited a local minimal value under all loading conditions. Thus, the serpentine discrete-stress distribution of the entire continuous beam partitioned into several independent intervals in the remainder of the arc. This stress discretisation indicates that the bending of arcs dominates the deformation of our ‘ribbon’ and allows the serpentines to be modified unrestrictedly to achieve additional functions. We instantiated this function as an irreversible connection by adding a cylindrical feature along the central axis of the two stacked ASCB-based structures (Fig. 5a).

In conclusion, an appropriate α guarantees that the two connected ASCB-nRs are free of interference, even when unstable deformation occurs. Additionally, the combination of serpentines and arcs endows the entire structure with stackability without sacrificing mechanical performance. Next, we exploited these two features to develop modules for our architected materials.

### 3.2 Design of 2D 3D modules

Three essential requirements must be satisfied to develop stackable modules. First, for all modules, regardless of their dimensions, the junctions for assembly should be the same, and the connections between them should be reversible. Second, the addition of junctions to the original structure should not adversely affect the mechanical performance significantly. Third, the dock surfaces between the modules should remain planar when the assembled structure is deformed. We demonstrated that the stress discretisation of ASCB-nR can potentially fulfil the second requirement. In this section, we provide a derivative design principle for developing modules from ASCB-nR and solutions for the remaining requirements.

**Fig. 4:**
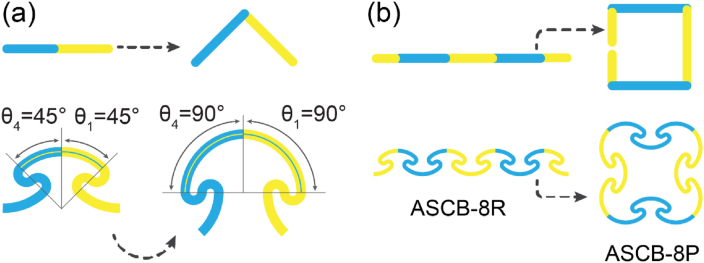
The schematic view of constructing a square (ASCB-8P) by eight ASCBs. (a) Bending a straight line consisting of two ASCBs as a right-angle by changing the values of central angles of the specific arcs. (b) Wrapping a straight ribbon (ASCB-8R) into a square frame (ASCB-8P).

The equal values of central angles of all the arcs causes an ASCB-nR to appear as a straight line. Thus, changing the specific central angles forms a corner, as shown in Fig. 4a. Furthermore, we contained ASCB-8R in a square by altering eight central angles from 45° to 90° (Fig. 4b). We refer to this square as ASCB-8P, where ‘P’ indicates that the topology of this object is homotopic to that of a polygon.

**Fig. 5:**
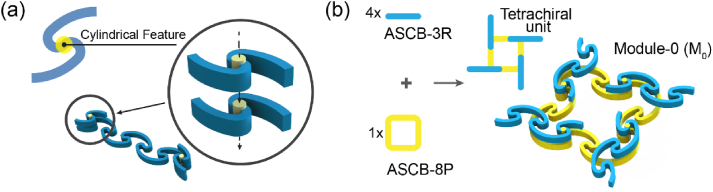
The design principles of Module-0. (a) The irreversible stacking of two ASCB via a cylindrical feature locating through their centers. (b) The scheme of combining four ASCB-3R and one ASCB-8P as a Module-0 by irreversible stacking.

We irreversibly stacked four ASCB-3Rs on ASCB-8P as a tetrachiral four-arm structure and named it as Module-0 (denoted as M_0_, Fig. 5b); its handedness is described in the Supplementary Materials. M_0_ is the basis for the subsequent 2D and 3D modules. We investigated the behaviour of M_0_ under uniaxial compression loads exerting along one or two orthogonal axes via FEA. The results showed that the axial load sheared and rotated the ASCB-8P of M_0_ owing to the non-zero resultant force and moment (Fig. S1b). The two axial loads cancelled the resultant force of M_0_ such that the central square exhibited an approximately rigid rotation (Fig. S1a).

**Fig. 6:**
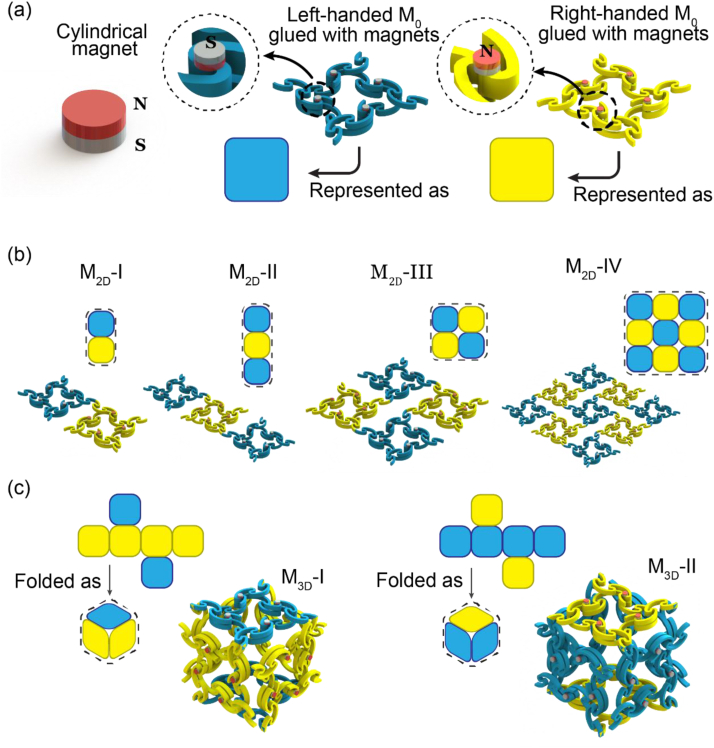
The schematic view of deriving the series of two-dimensional module (M_2D_) and three-dimensional module (M_3D_). (a) Gluing four cylindrical magnets on a M_0_. We simplified the left-handed M_0_ glued with magnets as a blue square, and the right-handed one as a yellow square. (b) Four types of 2D modules (M_2D_). (c) Folding two types of tessellations as 3D modules (M_3D_).

We designed four types of 2D modules by composing M_0_s in 1 × 2, 1 × 3, 2 × 2, and 3 × 3 staggered orders of antitetrachiral auxetic mechanical metamaterials [[31], [32], [33]] and denoted them as M_2D_-I, M_2D_-II, M_2D_-III, and M_2D_-IV, respectively (Fig. 6b). Additionally, as shown in Fig. 6c, we folded the two types of tessellations into cubes with six M_0_s as a 3D module and denoted it as M_3D_. We configured the handedness of M_0_ located at the top and bottom facets of the cube as opposite to that of the four lateral M_0_. This handedness configuration ensured the mechanical stability of M_3D_. We refer to the 3D modules whose top faces were left-handed/right-handed as M_3D_-I/M_3D_-II, respectively (Fig. 6c).

We analysed the responses of M_3D_ to shear, bending, twisting, and uniaxial compression/stretching via FEA. The results showed that the ‘ribbons’ (ASCB-3R) rotated or sheared their connected square (ASCB-8P) for all the loading cases (Fig. S2). The curved deformations of six ASCB-8Ps were negligible. This non-curving feature benefitted from the in-planar deformation mechanism of the connected ASCB-3Rs and qualified M_0_ as the docking surface. Hence, we adhered cylindrical magnets to each cylindrical feature of all M_0_s (Fig. 6a). These magnets provided general reversible junctions for mechanical assembly. In conclusion, we proposed M_0_ and magnetic stacking as solutions to the first and third requirements.

### 3.3 Design of modules variants

**Fig. 7:**
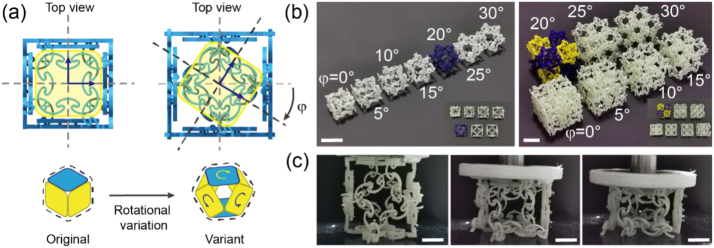
The design principles and samples of the variants of M_3D_. (a) Generating the variant of M_3D_ by mimicking the twisting deformation of the original one. (b) From left to right, seven samples of variants (single module and 2 × 2 × 2 assembled materials) whose rotating angle φ ranging from 0° to 30°. The image at bottom right corner is the top view of the main image. The length of scale bar represents 36 mm. (c) From left to right, The realistic view of the elastic deformation of a variant (φ = 15°) when its compressed strain is zero, threshold and maximum. The length of scale bar represents 10 mm.

To expand the design space, we introduced a non-scaling strategy (more details are provided in the Supplementary Materials) to evaluate the variants of the standard modules. Based on observation, the twisting action on the two quadra-connected M_0_s of M_3D_ triggered volumetric expansion or shrinkage, depending on the load direction (Fig. S2). These twisted states were transformed into the initial free states of the variants of M_2D_ and M_3D_ (Fig. 7a). Specifically, we rotated the ASCB-8Ps of the modules by an angle of φ (whose value was 0°,5°, …, 30°, as shown in Fig. 7b) around their geometric centre and recalculated the central angles of all the arc sections of ASCB-6R/ASCB-3R that were affected by this rotation (Fig. 7a) using a heuristic algorithm (more details are available in the Supplemental Materials). To achieve a larger volume of the variants, the rotation direction of ASCB-8P of the left-handed M_0_ unit was clockwise, and that of the right-handed M_0_ unit was counter-clockwise. Although this strategy was developed based on M_3D_, it is applicable to M_2D_ because these two series of modules were derived from M_0_.

**Fig. 8:**
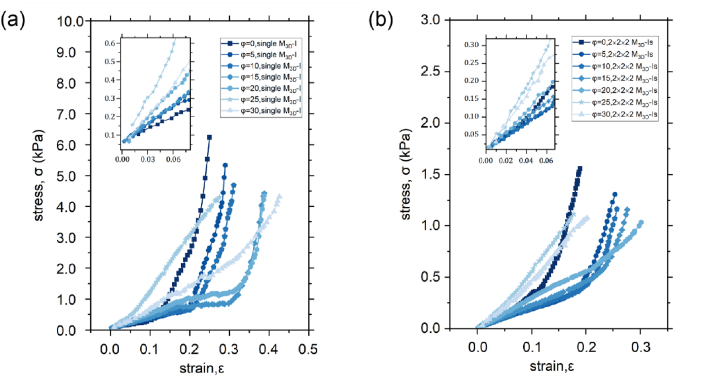
The stress-strain curve of the samples. (a) Single variants. (b) 2 × 2 × 2 assembled materials.

We experimentally investigated the individual (single voxel, as shown in Fig. 8a) and collaborative (2 × 2 × 2 assembled materials, as shown in Fig. 8b) behaviours of these seven variants under uniaxial compression exerting on quadra-connected M_0_s. The results showed that these variants exhibited two types of behaviour, which are explained as follows (Fig. 8):

1) The variants whose φ value was within the range of 0°–20° exhibited a low and almost constant strength (soft stage) until their compressed strains reached the threshold. Subsequently, the stress–strain curves ascended significantly (stiff stage). 2) The stress–strain relationships of the variants whose φ values exceeded 20° were both approximately linear. We treated the samples as homogeneous and denoted the Young's modulus of the first type of variants in the initial soft stage as $\bar{E_{s}}$ and the threshold strain as $\varepsilon^{*}$. For both cases of individual and assembled materials, the $\bar{E_{s}}$ and $\varepsilon^{*}$ increased proportionally with φ. Consequently, the variants with φ = 20° indicated higher values of $\bar{E_{s}}$ (4.68 and 2.90 kPa) and $\varepsilon^{*}$ (0.31 and 0.2) than the other variants with smaller φ (Table 1, Table 2). These results can be explained as follows: A larger φ provides more space for M_0_s to be rotated and sheared, thus resulting in an increase in $\varepsilon^{*}$. Moreover, the larger φ decreases the length (denoted as $l_{r}$) of the arm vector of the couple rotating the four bi-connected M_0_s, thus rendering them susceptible to being sheared than rotated; consequently, more elastic energy is absorbed and $\bar{E_{s}}$ is increased. Furthermore, the $l_{r}$ of the variants with φ > 20° was approximately zero, which implied that the handedness of M_0_ was transformed into axisymmetric along the load axis. This geometric phase transition rendered the Poisson's ratio of these variants positive and their effective stiffness approximately constant.

**Table 1:** Geometric parameters and mechanical performance of five variants of module.

Parameters	Values				
**Variant angle (°)**	0°	5°	10°	15°	20°
**Area (mm**^**2**^**)**	1.30 × 10^3^	1.53 × 10^3^	1.72 × 10^3^	1.80 × 10^3^	1.96 × 10^3^
**Height (mm)**	36.00	38.00	39.6	41.00	42.10
**Effective Young's Modules in soft stage (kPa)**	3.23	3.50	3.69	3.85	4.68
**Threshold strain (1)**	0.10	0.18	0.20	0.25	0.31

**Table 2:** Geometric parameters and mechanical performance of five 2 × 2 × 2 variants of architected materials.

Parameters	Values				
**Variant angle (°)**	0°	5°	10°	15°	20°
**Area (mm**^**2**^**)**	5.22 × 10^3^	6.12 × 10^3^	6.89 × 10^3^	7.19 × 10^3^	7.82 × 10^3^
**Height (mm)**	72.00	76.00	79.2	82.00	84.20
**Effective Young's Modules in soft stage (kPa)**	2.42	1.88	1.95	2.25	2.90
**Threshold strain (1)**	0.04	0.16	0.17	0.18	0.20

In conclusion, our optimal strategy expanded the design space of ASCB-based modules of various sizes, tunable stiffnesses ranging over one order of magnitude, as well as endowed flexible adaptability for different scenarios. Without loss of generality, we selected the series of the modules whose φ = 20° (including M_2D_ and M_3D_) to construct our RAMs for the following loading support implementations.

### 3.4 Construction of RAMs

**Fig. 9:**
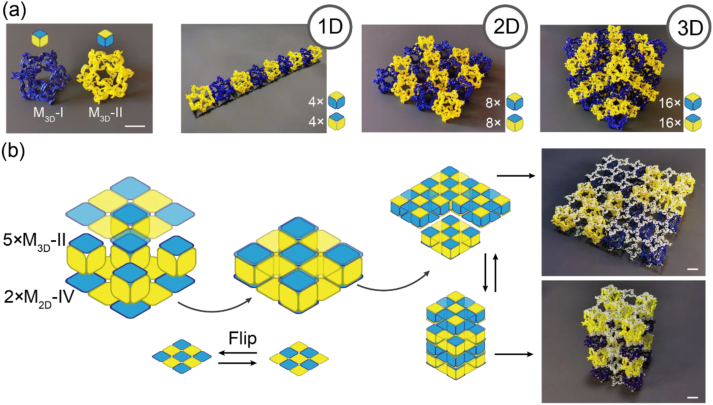
Regular assembled architected materials. (a) Two colored M_3D_ (φ = 20°). The rest from left to right are 1D (1 × 8), 2D (4 × 4) and 3D (4 × 4 × 4) assembled architected materials. (c) The scheme of assembling a “sandwich” hybrid cell consisting of five M_3D_s and two M_2D_-IVs. The assembled materials can be recycled between different shapes, such as 2 × 2 (shell)and 1 × 4 (pillar). Flipping M_2D_-IV over alters its handedness.

The fundamental principle of assembling a handed module is that the handedness configuration on a pair of stacked M_0_ units should be opposite to avoid internal geometric frustration [2,4]. To illustrate the structure efficiently, we colour coded M_3D_-I and M_3D_-II with blue and yellow, respectively (Fig. 9a). As three connectable axes were present in one M_3D,_ the axes can be utilised individually to develop series of architected materials ranging from one dimensional to 3D. For example, we assembled three types of materials measuring 1 × 8 (rod sample), 4 × 4 (sheet sample), and 4 × 4 × 4 (bulk sample). Moreover, unlike conventional architected materials featuring one type of cell, our materials had compatible docking surfaces, M_0_, for merging 2D and 3D modules, thus expanding the assembly type of our RAMs. We assembled a hybrid cell comprising five M_3D_s and two M_2D_-IVs (Fig. 9b). The docking faces of the M_2D_-IVs were the central and corner M_0_. The five M_3D_s were connected to the M_2D_-IVs through these faces via their top and bottom faces, thus resulting in a sandwich structure. Notably, flipping the M_2D_-IVs inverted the handedness of M_0_s. Consequently, we defined the handedness of this hybrid cell to be the same as that of its M_3D_s. We spanned this cell into a 2 × 2 plate and 1 × 4 column by implementing stacking between the M_3D_s and M_2D_-IVs, respectively (Fig. 9b). Moreover, we transformed these two types of architected materials by exchanging the stacked modules. Thus, we used 8 M_2D_s to replace 16 M_3D_s and maintained approximately the same volume of the architected material assembled using M_3D_s alone.

**Fig. 10:**
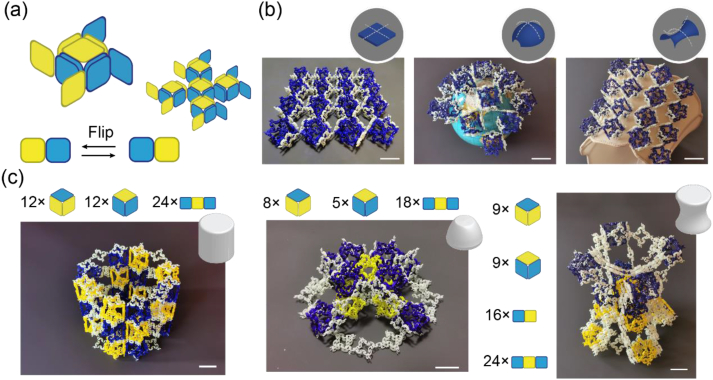
Curved assembled architected materials. (a) The schematic view of assembling 4 × 4 flexible architected shell consisting of chiral hybrid units. (b) From left to right, this material is flexible and adaptive to the surfaces whose Gaussian curvature equal to zero, positive and negative. (c)From left to right are barrel, convex hull and vase which are consisted of four kinds of modules. The length of scale bar represents 36 mm.

In addition to this shell-shaped hybrid cell, we provided a chiral hybrid cell to expand the potential of our scheme for merging modules at the cell level. We stacked four M_2D_-Is onto the lateral M_0_s of one M_3D_, thus resulting in a 3D chiral unit (Fig. 10a). We observed that each M_2D_-I offered only one ASCB-6R connecting two adjacent hybrid chiral units, which significantly decreased the bending stiffness of the shell structure and rendered it soft and flexible, which is analogous to a mass–spring multibody dynamic system such as cloth. The materials were attached to different surfaces with Gaussian curvatures ranging from negative to positive (Fig. 10b). Additionally, we constructed complex structures with non-zero initial curvatures using this merging assembly. Analogous to the abovementioned ‘sandwich’ hybrid cell, we bridged two M_2D_-IIs (1 × 3) over the top and bottom of two M_3D_s and allowed their centre M_0_s to be free. Subsequently, we expanded this hybrid pillar cell into a 3 × 4 architected material and encapsulated it to form a cylinder (Fig. 10c and MovieS1). Moreover, we stacked one end of the four pillar cells on the four lateral M_0_s of one M_3D_ and constrained the other four free ends using a thin ring comprising eight M_2D_-IIs. The interior was filled with four M_3D_s and two M_2D_-IIs to form a convex hull (Fig. 5b). Finally, we altered the connecting M_0_s between the four cuboids and central cube, and transformed the convex surfaces into concave surfaces. With the assistance of the constraint ring (comprising eight M_2D_-Is) stacked inside, we constructed a ‘vase’ (Fig. 10c). The Gaussian curvatures of these three shapes ranged from negative to positive, thus indicating that our modules and stacking mechanism can potentially be used to construct complex structures with curved shapes. The M_2D_s provide flexibility to these curved structures, whereas the M_3D_s provide partial load support.

### 3.5 Applications of RAMs

**Fig. 11:**
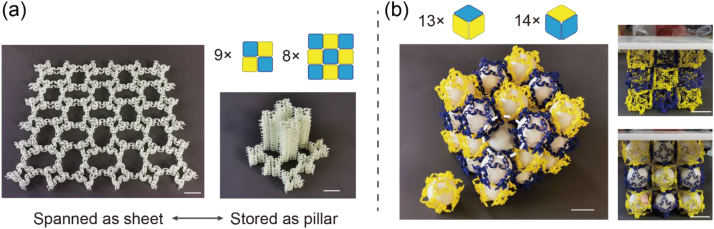
Free-damaged recyclable sheet and mechanical tunable bulk architected materials. (a) A soft sheet-like architected material assembled by 9 M_2D_-IIIs and 8 M_2D_-IVs was recycled and rebuilt as a rigid pillar. (b) A 3 × 3 × 3 architected material assembled by 13 M_3D_-Is and 14 M_3D_-IIs. We recycled it back to modules and fulfilled 31 elastic balls inside and between them. The reassembled and modified material showed more rigid behaviour than the original one. The length of scale bar represents 36 mm.

Typically, to store large-scale sheet-shaped materials whose thickness is at least one order of magnitude lower than the lengths in the other two dimensions, we must curl or fold them to conserve space. In the long term, such storage changes the initial curvature or results in undesired wrinkles in the sheets, thus deteriorating their quality and function. However, for recyclable materials, one can realise undamaged storage efficiently. For instance, we assembled a 6 × 6 flexible sheet comprising nine M_2D_-IIIs and eight M_2D_-IVs (Fig. 11a) and then recycled them into modules by disassembling and stacking them as rigid pillars. This reassembly intricately avoided unexpected curving or fold marks and improved storage-space utilisation.

Additionally, we exploited the hollowness of M_3D_ to demonstrate another recyclability benefit. In conventional reconfigurable materials, their shape or topology is typically changed to alter their effective strength. This requires a preset in the initial design stage andcomplex external stimuli. By recycling, our hollow 3D module provides a cost-effective and convenient method to increase the overall structural stiffness of the material. We assembled 3 × 3 × 3 bulk materials using 13 M_3D_-Is and 14 M_3D_-IIs. After recycling, rigid spheres were added to the interior of a single M_3D_ and to the gaps between multiple M_3D_s. Under a load that compressed the unfilled state by 7 %, the filled material exhibited near-zero deformation (Fig. 11b). Although the rigid balls used in this study were ping pong balls, they can be replaced by soft elastic sponge balls or hard metal balls to realise various stiffnesses (ranging several orders of magnitude) for the assembled architected materials.

**Fig. 12:**
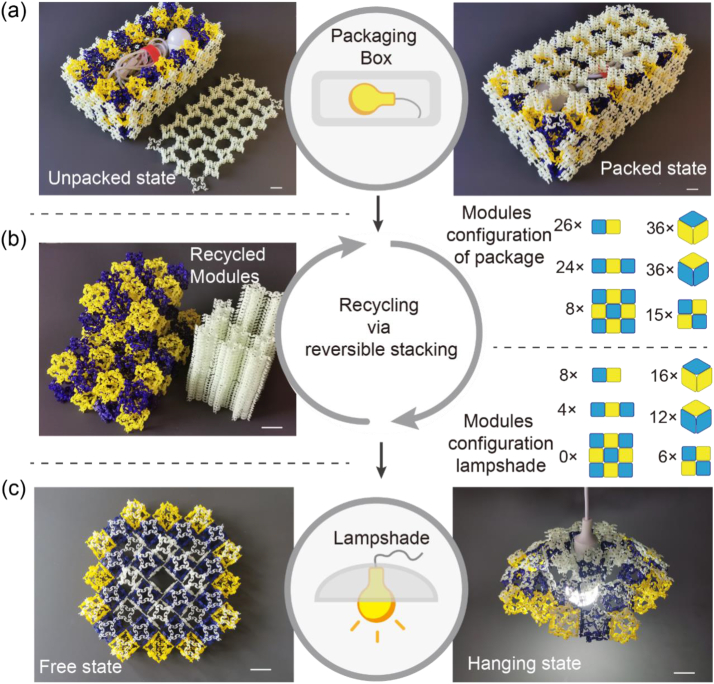
The demonstration of switching the function of an assembled package between protection and light-area controlling via in-situ mechanical recycling. (a) From left to right, an opened or closed packaging box containing a fragile bulb and cables. (b) Recycling this package and efficiently stored the dissembled modules in a smaller volume. (c) Reassembled those recycled modules to build a lampshade without extra tools and constructed a suspending light with the bulb. The length of scale bar represents 36 mm.

Fragile and sophisticated items must be placed in customised packaging boxes to avoid damage during transportation. However, in most cases, boxes were left unused after item removal. This waste of material and space must be considered in resource-constrained circumstances. In this study, we demonstrate the manner by which our recyclable architected materials can solve such problems. We assembled a 4 × 8 × 3 packaging box for supporting the fragile bulb and its cable. This box comprised 72 M_3D_s, 26 M_2D_-Is, 24 M_2D_-IIs, 15 M_2D_-IIIs, and 8 M_2D_-IVs (Fig. 12a). All M_3D_s were used to construct the main body of the box, including 12 for the bottom, 48 for the large lateral walls, and the remaining for small lateral walls. In addition, we assembled 16 M_2D_-Is and 15 M_2D_-IIIs as box covers. The remaining modules were used to strengthen the walls. Subsequently, the cover was opened and the bulbs and cables were removed. Analogous to the recycling of paper or glass, we disassembled the box into its modules, which served as raw materials, as shown in (Fig. 12b). Next, we reassembled the soft hybrid materials (Fig. 10a) using 12 M_2D_-Is and 12 M_2D_-IIIs and closed their edges with 12 M_2D_-IIs. Moreover, we added an additional layer at the top to strengthen the effective rigidity of the entire structure and prevent it from collapsing. This strengthening layer comprised eight M_2D_-Is around the central hole, four M_2D_-IIIs at the corner, and four M_2D_-IIs (Fig. 12c and MovieS2). Next, the cable was inserted into the central hole and passed through the oval-shaped hole of the two stacked M_2D_-IIIs attached to the bottom of the structure. Finally, we suspended the cable, which rendered the composite structure resembles the curved structure of a lampshade (Fig. 12c).

## 4 Discussion

Thus far, we have introduced two types of stacking, i.e. irreversible and reversible stacking, to design and develop our ASCB-based RAMs. Benefitting from the stress discretisation of ASCB-nR/mP, we stacked ASCB-3Rs and ASCB-6Ps and fixed their relative positions by combining their cylindrical features in the design stage, wherein the mechanical performance was not affected by the adverse node effect. Those two types of ASCBs were combined in this irreversible stacking, thus resulting in a prototype module M_0_, which served as the constructive element and docking facet for the subsequent M_2D_ and M_3D_ modules. Furthermore, this stacking mechanism caused the connected pairs to undergo in-plane deformation under uniaxial loads, thus preventing geometric frustration from the interference between beams. We used magnets to provide joints for reversible stacking between the M_0_s, which is fundamental for realising recycling. Because one magnet comprises two poles, we adopted a chiral topology to construct the M_0_ with two handedness. We attached the south/north pole of the magnet on a left-/right-handed M_0_ module. This one-to-one mapping facilitated the manufacture of RAMs. In conclusion, these two stacking mechanisms ensured that the entire system was free of internal frustration and offered a stable and effective mechanical method for recycling. The heuristic method for designing variants of the M_3D_ module provided more freedom in the assembly process for selecting appropriate modules whose mechanical properties, including the effective stiffness and threshold compression strain, satisfy the requirements of specific scenarios. Owing to being derived from the same original module M_0_, the M_2D_ series of the modules share the same method for the design of comparable variants whose φ is the same as those of the M_3D_ modules. Notably, φ=20° mays not be ideal for all types of scenarios. For example, the variants with φ=0° are more suitable for assembling architected materials for use in space-limited and load-bearing applications owing to their smaller volumes and higher stiffness compared with the others.

Generally, the packaging box of a product is only an accessory that offers a protective role during transportation and does not constitute the product. However, recycling from a packaging box to a bunch of stacked raw materials and then adopting some of them to construct a lampshade is a sustainable example of the recycling of protective accessories of a chandelier; consequently, a lampshade is created to control the lighting area and enhance aesthetics. This on-site recycling not only minimises volumes and materials, but also improves sustainability and provides more space for creative designers.

Our magnet-stacking mechanism reverses the assembly of RAMs and decreases the maximum bearable value of stretching or shearing. Because compression only strengthens the stability of the magnetic connections, the RAMs maintain their internal connectivity as a monolithic material. However, unlike the maintenance of stability under increasing compression loads, magnetic junctions are susceptible to disconnection when the stretching loads exceed the limit of attractive forces between magnets. Although we can enlarge the size of the magnets to overcome this limitation, we realise that RAMs are yet to be optimised for supporting long-term stretching or hydrostatic loads, such as pneumatic inflatables and structural shells of spaceships or submarines.

## 5 Conclusion

Herein, we presented a category of ASCB-based modular architected materials that can be mechanically recycled in situ. The staggered order of the arcs and serpentines caused the ASCB to exhibit stress discretisation (stress distributed discretely over a continuous beam) under loads. These unreported properties provide ideal locations for adding reversible magnetic joints in ASCB-based modules without loss of mechanical performance. An ASCB with an appropriate α guarantees that its derivative modules are free of internal frustration [34,35]. These two key features provide a reversible stacking mechanism for our modules, thus allowing the assembly of RAMs that are adaptive to various sizes and curvatures, ranging from pure materials to composite structures, regular samples to complicated curved shapes, and flexible thick sheets to rigid bulk materials. Finally, we recycled the cuboid packaging box to its raw state and reconstructed a convex lampshade. Our RAMs were successfully recycled into various shapes, which included a complete spectrum of Gaussian curvatures, and afforded different mechanical properties and functions. Combining our findings with hierarchical design tools and automatic manufacturing, we believe that our study will promote economic and robust implementation in emerging fields, including reprogrammable materials [22,36,37], swarm and modular robotics [38,39], and modular architectural designs [40,41] for resource-limited circumstances such as inside closed rooms of off-road vehicles, submarines, or spaceships.

## Data availability statement

Data supporting the findings of this study is not deposited into any publicly available repository and is available from the corresponding author on request.

## CRediT authorship contribution statement

**Hongyi Yao:** Conceptualization, Data curation, Formal analysis, Investigation, Methodology, Visualization, Writing – original draft, Writing – review & editing. **Xiaoyu Zhao:** Conceptualization, Data curation, Formal analysis, Visualization. **Shengli Mi:** Conceptualization, Data curation, Formal analysis, Funding acquisition, Writing – review & editing.

## Declaration of competing interest

The authors declare that they have no known competing financial interests or personal relationships that could have appeared to influence the work reported in this paper.

## References

[1] Mhatre S., Boatti E., Melancon D., Zareei A., Dupont M., Bechthold M., Bertoldi K. (2021). Deployable structures based on buckling of curved beams upon a rotational input. Adv. Funct. Mater..

[2] Jenett B., Cameron C., Tourlomousis F., Rubio A.P., Ochalek M., Gershenfeld N. (2020). Discretely assembled mechanical metamaterials. Sci. Adv..

[3] Kim J.Z., Lu Z., Strogatz S.H., Bassett D.S. (2019). Conformational control of mechanical networks. Nat. Phys..

[4] Frenzel T., Kadic M., Wegener M. (2017). Three-dimensional mechanical metamaterials with a twist. Science.

[5] Berger J.B., Wadley H.N., McMeeking R.M. (2017). Mechanical metamaterials at the theoretical limit of isotropic elastic stiffness. Nature.

[6] Melancon D., Gorissen B., Garcia-Mora C.J., Hoberman C., Bertoldi K. (2021). Multistable inflatable origami structures at the metre scale. Nature.

[7] Kamrava S., Ghosh R., Xiong J., Felton S.M., Vaziri A. (2019). Origami-equivalent compliant mechanism. Appl. Phys. Lett..

[8] Dieleman P., Vasmel N., Waitukaitis S., van Hecke M. (2019). Jigsaw puzzle design of pluripotent origami. Nat. Phys..

[9] Overvelde J.T., Weaver J.C., Hoberman C., Bertoldi K. (2017). Rational design of reconfigurable prismatic architected materials. Nature.

[10] Overvelde J.T., de Jong T.A., Shevchenko Y., Becerra S.A., Whitesides G.M., Weaver J.C., Hoberman C., Bertoldi K. (2016). A three-dimensional actuated origami-inspired transformable metamaterial with multiple degrees of freedom. Nat. Commun..

[11] Dudte L.H., Vouga E., Tachi T., Mahadevan L. (2016). Programming curvature using origami tessellations. Nat. Mater..

[12] Li J., Ran R., Wang H., Wang Y., Chen Y., Niu S., Arratia P.E., Yang S. (2021). Aerodynamics-assisted, efficient and scalable kirigami fog collectors. Nat. Commun..

[13] Rafsanjani A., Jin L., Deng B., Bertoldi K. (2019). Propagation of pop ups in kirigami shells. Proc. Natl. Acad. Sci. U.S.A..

[14] Choi G.P.T., Dudte L.H., Mahadevan L. (2019). Programming shape using kirigami tessellations. Nat. Mater..

[15] Rafsanjani A., Bertoldi K. (2017). Buckling-Induced kirigami. Phys. Rev. Lett..

[16] Cho Y., Shin J.H., Costa A., Kim T.A., Kunin V., Li J., Lee S.Y., Yang S., Han H.N., Choi I.S., Srolovitz D.J. (2014). Engineering the shape and structure of materials by fractal cut. Proc. Natl. Acad. Sci. U.S.A..

[17] van Manen T., Janbaz S., Ganjian M., Zadpoor A.A. (2020). Kirigami-enabled self-folding origami. Mater. Today.

[18] Li S., Deng B., Grinthal A., Schneider-Yamamura A., Kang J., Martens R.S., Zhang C.T., Li J., Yu S., Bertoldi K., Aizenberg J. (2021). Liquid-induced topological transformations of cellular microstructures. Nature.

[19] Chen T., Pauly M., Reis P.M. (2021). A reprogrammable mechanical metamaterial with stable memory. Nature.

[20] Xia X., Afshar A., Yang H., Portela C.M., Kochmann D.M., Di Leo C.V., Greer J.R. (2019). Electrochemically reconfigurable architected materials. Nature.

[21] Jackson J.A., Messner M.C., Dudukovic N.A., Smith W.L., Bekker L., Moran B., Golobic A.M., Pascall A.J., Duoss E.B., Loh K.J., Spadaccini C.M. (2018). Field responsive mechanical metamaterials. Sci. Adv..

[22] Zhang Y., Velay-Lizancos M., Restrepo D., Mankame N.D., Zavattieri P.D. (2021). Architected material analogs for shape memory alloys. Matter.

[23] La Porta C.A.M., Lionetti M.C., Bonfanti S., Milan S., Ferrario C., Rayneau-Kirkhope D., Beretta M., Hanifpour M., Fascio U., Ascagni M., De Paola L., Budrikis Z., Schiavoni M., Falletta E., Caselli A., Chepizhko O., Tuissi A., Vailati A., Zapperi S. (2019). Metamaterial architecture from a self-shaping carnivorous plant. Proc. Natl. Acad. Sci. U.S.A..

[24] Xin L., Siyuan Y., Harry L., Minghui L., Yanfeng C. (2020). Topological mechanical metamaterials: a brief review. Curr. Opin. Solid State Mater. Sci..

[25] Meeussen A.S., Oguz E.C., Shokef Y., van Hecke M. (2020). Topological defects produce exotic mechanics in complex metamaterials. Nat. Phys..

[26] Zhang Y., Li B., Zheng Q.S., Genin G.M., Chen C.Q. (2019). Programmable and robust static topological solitons in mechanical metamaterials. Nat. Commun..

[27] Liu B., Silverberg J.L., Evans A.A., Santangelo C.D., Lang R.J., Hull T.C., Cohen I. (2018). Topological kinematics of origami metamaterials. Nat. Phys..

[28] Waitukaitis S., van Hecke M. (2016). Origami building blocks: generic and special four-vertices. Phys. Rev. E.

[29] Nezerka V., Somr M., Janda T., Vorel J., Doskar M., Antos J., Zeman J., Novak J. (2018). A jigsaw puzzle metamaterial concept. Compos. Struct..

[30] Li Y.B., Zhang Q.T., Hong Y.Y., Yin J. (2021). 3D transformable modular kirigami based programmable metamaterials. Adv. Funct. Mater..

[31] Wu W., Hu W., Qian G., Liao H., Xu X., Berto F. (2019). Mechanical design and multifunctional applications of chiral mechanical metamaterials: a review. Mater. Des..

[32] Surjadi J.U., Gao L., Du H., Li X., Xiong X., Fang N.X., Lu Y. (2019). Mechanical metamaterials and their engineering applications. Adv. Eng. Mater..

[33] Kolken H.M.A., Zadpoor A.A. (2017). Auxetic mechanical metamaterials. RSC Adv..

[34] Coulais C., Teomy E., de Reus K., Shokef Y., van Hecke M. (2016). Combinatorial design of textured mechanical metamaterials. Nature.

[35] Bertoldi K., Vitelli V., Christensen J., van Hecke M. (2017). Flexible mechanical metamaterials. Nat. Rev. Mater..

[36] Lendlein A., Gould O.E.C. (2019). Reprogrammable recovery and actuation behaviour of shape-memory polymers. Nat. Rev. Mater..

[37] Ze Q., Kuang X., Wu S., Wong J., Montgomery S.M., Zhang R., Kovitz J.M., Yang F., Qi H.J., Zhao R. (2020). Magnetic shape memory polymers with integrated multifunctional shape manipulation [article]. Adv. Mater..

[38] Peng Z., Jiang Y., Wang J. (2021). Event-triggered dynamic surface control of an underactuated autonomous surface vehicle for target enclosing [article]. IEEE Trans. Ind. Electron..

[39] Brambilla M., Ferrante E., Birattari M., Dorigo M. (2013). Swarm robotics: a review from the swarm engineering perspective [Article]. Swarm Intelligence.

[40] Soegoto E.S., Subarjat R., Valentina T. (2019).

[41] Mlcochova M., Pavel R. (2016). The future of architecture in modular construction [Journal Paper]. Appl. Mech. Mater..

