# Supplementary material for: Modular design of curved beam-based recyclable architected materials
Source: Heliyon. 2023 Nov 7;9(11):e21557. doi: 10.1016/j.heliyon.2023.e21557 (PMC10694173; doi:10.1016/j.heliyon.2023.e21557)
Supplement: Multimedia component 1 [file mmc1.pdf]

Supplementary Information

Modular Design of Curved Beam-Based Recyclable Architected Materials

Authors

Hongyi Yao[1,2], Xiaoyu Zhao[1] and Shengli Mi[1,2*]

Affiliations

1. Bio-manufacturing Engineering Laboratory, Tsinghua Shenzhen International Graduate School, Tsinghua University, Shenzhen, China.
2. Department of Mechanical Engineering, Tsinghua University, Beijing, China.

*: Corresponding author
E-mail: mi.shengli@sz.tsinghua.edu.cn
Bio-manufacturing Engineering Laboratory
Tsinghua Shenzhen International Graduate School, Tsinghua University
Rm 1112, energy and environment Building, Tsinghua Campus,
The University Town, Shenzhen 518055, P.R. China

1.MATHEMATICA MODEL OF GENERAL ASC

An individual ASC is consisted of 4 arcs. First, we locate the centers of these arcs $A_j^0\ (i=1,2...4)$ at the origin of a global coordinate system as:

$$A_j^0(t_j) = \rho_j e^{i(\varphi_j + sign(\frac{5}{2}-j)t_j)} \tag{1}$$

where ρ_i and $\varphi_{i,a}$ are the radius and phase angle of the jth arc, respectively. Besides, φ_i among four arcs satisfy a recursive relation:

$$\varphi_{j+1} = \varphi_j + \alpha_j\theta_j - \frac{(1-\alpha_j\alpha_{j+1})}{2}\pi \tag{2}$$

where we set $|\varphi_1| = \frac{\pi}{2}$, and α_j is the sign of the curvature of A_j^0 .It is worth noting that the signs of φ_1 and α_1 is opposite. Moreover, t_j is a parameter which indicates A_j^0 as the trajectory of a point moving from one end of the j th arc to another, ranging from zero to θ_i , the central angle of the i th arc. Then, we translate and combine these arcs from head to tail tangentially by imposing the recursive relation

$$A_{j+1}(t_j) = A_{j+1}^0(t_j) - A_{j+1}^0(0) + A_j^0(\theta_j) \tag{3}$$

where $A_j(t_j)$ is the translated ith arc. Finally, we sum these trajectories up and get the curve model as

$$G(t) = \sum_{j=1}^{4}\delta_{jl}A_j(t - \sum_{k=1}^{l}\theta_k) \tag{4}$$

where $t \in [0, \sum_{j=1}^{4}\theta_j]$, $l = 5 - \sum_{j=1}^{4}\left\lfloor \frac{min\{\sum_{k=1}^{j}\theta_k, t\}}{t} \right\rfloor$.

It is noted that $\sum_{j=2}^{3}\delta_{jl}A_j(t - \sum_{k=1}^{l}\theta_k)$ describes the shape of serpentine. It is worth noting that ρ_i^0 , θ_i^0 and φ_1 determine the free-state shape of ASC. The angle between the velocity vector of ASC at $G(0)$ and $G(\sum_{j=1}^{4}\theta_j)$ is

$\theta_c = \sum_{i=1}^{4} \alpha_i \theta_i$. For convenience, we define a standard left-handed ASC whose $\varphi_1 = -\frac{\pi}{2}$, ρ_k^0 and θ_i^0 satisfy a group of symmetric conditions

$$\begin{cases} \theta_2^0 = \theta_3^0 \\ \theta_k^0(k<2) = \theta_l^0(l>2) \\ \rho_k^0(k<2) = \rho_l^0(l>2) \\ \rho_2^0 = \rho_2^0 = \frac{\rho_1^0}{4}(2-\sec\theta_1^0) \\ \alpha_k(k<2) = -\alpha_l(l>2) = 1 \end{cases} \tag{5}$$

For numerical simulation, we change t_j into a discrete form as

$$t_j = \frac{m_j \theta_j}{n_j - 1}, m_j = 0, 1, \ldots n_j - 1 \tag{6}$$

where n_j is the number of sample nodes locating at the i th arc. In order to unify the distances between nodes distributed along the whole curve of ASC, we normalize n_i as

$$n_j = \left\lfloor n_1 \frac{\rho_j \theta_j}{\rho_1 \theta_1} \right\rfloor \tag{7}$$

So, the discrete form of equation is updated as

$$\hat{G}(m) = \sum_{j=1}^{4} \delta_{jl} \hat{A}_j(\beta) \tag{8}$$

where $m \in [0, \sum_{j=1}^{4} n_j - 3]$, $\beta = \frac{\left(m - \sum_{k=1}^{l} n_k\right)\theta_j}{n_j - 1}$ and $l = 5 - \sum_{j=1}^{4} \left\lfloor \frac{min\{\sum_{k=1}^{j} n_k, m\}}{m} \right\rfloor$.

The geometric parameters of standard ASC are:

$$\begin{cases} \theta_2 = \theta_3 = \pi \\ \theta_1 = \theta_4 = \frac{\pi}{4} \\ \rho_1 = \rho_4 = 4 \\ \rho_2 = \rho_3 = \frac{\rho_1}{4}(2-\sec\theta_1) = 2-\sqrt{2} \end{cases} \tag{9}$$

and $G(0) = \mp 4i$ for left-handed and right-handedness, respectively. Consequently, the θ_c of standard ASC is zero. We also derived another ASC whose $\theta_c = \mp\frac{\pi}{4}$ by setting its θ_4 (θ_1) as $\frac{\pi}{2}$. The corresponding point object derived from standard ASC and this varied ASC is denoted as P and P'.

2.THE VOLUMETRIC EXPANSION FROM ASC TO ASCB

This operation expands ASC with a cross-section. To avoid lateral buckling and visualize twisting effect of P , we choose rectangle as the shape of cross-section. First, we establish an orthogonal coordinate system $\mathbf{F}^3$:

$$\mathbf{F}^3 = \left\{ \left(x_1, ix_2, x_3 \right) : x_j \in \mathbf{R} \; for \; j = 1,2,3 \right\} \tag{10}$$

where $i = \sqrt{-1}$ which turns the second real coordinate into a complex one. The directions of the three axis ($\tilde{e}_x, \tilde{e}_y, \tilde{e}_z$) control the normal, width and thickness orientation of the local cross-section, respectively. First, we compact the points of discrete ASC in a $m_{\max} \times 3$ matrix $\boldsymbol{G}^0$, where $m_{\max} = \sum_{j=1}^{4} n_j - 3$:

$$\boldsymbol{G}^0 = \begin{bmatrix} \mathbf{Re}\left(\hat{g}(0)\right) & \mathbf{Im}\left(\hat{g}(0)\right) & 0 \\ \mathbf{Re}\left(\hat{g}(1)\right) & \mathbf{Im}\left(\hat{g}(1)\right) & 0 \\ \vdots & \vdots & \vdots \\ \mathbf{Re}\left(\hat{g}(m_{\max})\right) & \mathbf{Im}\left(\hat{g}(m_{\max})\right) & 0 \end{bmatrix} \tag{11}$$

By implying forward difference, we calculate the normal vector of cross-section on the i th point as the i th row of matrix $\boldsymbol{R}^n_{i\bullet}$:

$$\boldsymbol{R}^n_{i\bullet} = \boldsymbol{G}^0_{(i+1)\bullet} - \boldsymbol{G}^0_{(i)\bullet}, i = 1, \ldots m_{\max} - 1 \tag{12}$$

Then, we characterized the rotation from $\boldsymbol{e}_1 = \left(1,0,0\right)$, the direction of global x_1 axis, to $\tilde{\boldsymbol{e}}^i_n = \frac{\boldsymbol{R}^n_{i\bullet}}{\left|\boldsymbol{R}^n_{i\bullet}\right|}$ as:

$$\begin{cases} \phi = \cos^{-1}(\boldsymbol{e}_1 \cdot \tilde{\boldsymbol{e}}^i_n) \\ \boldsymbol{z}_i = \boldsymbol{e}_1 \times \tilde{\boldsymbol{e}}^i_n \end{cases} \tag{13}$$

Which can be compacted the above as a $1\times(m_{\max} - 1)$ quaternion matrix Q^0 where

$$Q^0_i = \left(\cos\left(\frac{\phi}{2}\right), \sin\left(\frac{\phi}{2}\right)\boldsymbol{z}_i \right), i = 1, \ldots n-1 \tag{14}$$

Since Q represents the average value of the rotation of the coordinate axis at every two adjacent points, we need to solve the rotation at each point by the spherical linear interpolation(slerp) and get a $1\times m_{\max}$ quaternion matrix Q as

$$\begin{cases} Q_i = \dfrac{\sin(0.25\phi)}{\sin(0.5\phi)} Q^0_{i-1} + \dfrac{\sin(0.25\phi)}{\sin(0.5\phi)} Q^0_{i+1}, i = 2, \ldots m_{\max} - 1 \\ Q_1 = \dfrac{\sin(0.5\phi)}{\sin(0.25\phi)} \left(Q_2 - \dfrac{\sin(0.25\phi)}{\sin(0.5\phi)} Q_3 \right) \\ Q_m = \dfrac{\sin(0.5\phi)}{\sin(0.25\phi)} \left(Q_{m-1} - \dfrac{\sin(0.25\phi)}{\sin(0.5\phi)} Q_{m-2} \right) \end{cases} \tag{15}$$

Finally, we get the width and thickness vector of cross-section $R_{i\cdot}^{w}$ and $R_{i\cdot}^{h}$ through rotating from $\boldsymbol{e}_2 = (0,1,0)$ and $\boldsymbol{e}_3 = (0,0,1)$ by Q , respectively.

$$\begin{cases} \boldsymbol{R}_{i\cdot}^{w} = Q_i \ (1, \boldsymbol{e}_2) Q_i^* \\ \boldsymbol{R}_{i\cdot}^{t} = Q_i \ (1, \boldsymbol{e}_3) Q_i^* \end{cases} \tag{16}$$

We set the point on ASC as the origin of each local coordinate system. The vertices locating at each corner can be packed up and calculated as

$$\boldsymbol{G}^k = \boldsymbol{G}^0 + \frac{(-1)^k W}{2} \boldsymbol{R}^w + \frac{sign(2.5-k)T}{2} \boldsymbol{R}^t \tag{17}$$

where $G^k (k = 1,2,3,4)$ packing up the vertices locating at inner top corner, outer top corner, inner bottom corner and outer bottom corner, respectively. $W = 2\sqrt{2}$ and $T = 4\sqrt{2}$ are the width and thickness of the cross-section, respectively. Finally, we attach skins on these vertices by common triangulation algorithm to fulfill a P .

We define $\boldsymbol{M}^P$,a $5m_{\max} \times 3$ matrix of P , as:

$$\boldsymbol{M}^P = \begin{bmatrix} \boldsymbol{G}^0 \\ \boldsymbol{G}^1 \\ \boldsymbol{G}^2 \\ \boldsymbol{G}^3 \\ \boldsymbol{G}^4 \end{bmatrix} \tag{18}$$

In summary, we define P as a set whose elements are $\boldsymbol{M}^P$ and Q as:

$$P = \{\boldsymbol{M}^P, Q\} \tag{19}$$

3.THE WORKFLOW OF GENERATING ASCB-nR/mP

This procedure generates two kinds of L, thus, L_{nO} and L_{mC}. The notation L_{nO} denotes an ASCB-nR consisted of n P s with alternative handedness. Similarly, the notation L_{mC} denotes an ASCB-mP. From the above sections, we know there are eleven parameters to describe a point object. For convenience, we choose φ_1, θ_1 and θ_4 as variables and define a function f_P as:

$$P = f_P\left(\varphi_1, \theta_1, \theta_4 \left|\Theta\right.\right), \Theta = \left(\rho_1, \rho_2, \rho_3, \rho_4, \theta_2, \theta_3, W, T\right) \tag{20}$$

For a L_{nO} with a shape of straight line:

Step1: Generate standard P s with given number n and alternative handedness as:

$$P_i = f_P\left(\beta\varphi_1, \beta\theta_1, \beta\theta_4 \left|\Theta\right.\right), i = 1, \ldots n \tag{21}$$

where $\beta = \left(-1\right)^{i-1}\beta^*$, $\varphi_1 = -\frac{\pi}{2}$, θ_1, θ_4 and Θ satisfy the standard geometric conditions . We define a left-handedness L_{nO} whose $\beta^* = 1$ and verse vice.

Step2: Connect the above n P_i s in turn end to end, resulting as a L_{nO}. We translate P_{i+1} so that its head vertex coincides with the end vertex of P_i. The i th translation vector V_t^i is

$$V_t^i = \tilde{M}_{m_{\max}^i \bullet}^{P_i} - M_{1\bullet}^{P_{i+1}}, i = 1, \ldots n \tag{22}$$

where $M_{1\bullet}^{P_{i+1}}$ is the coordinates of the head vertex of P_{i+1} and $\tilde{M}_{m_{\max}^i \bullet}^{P_i}$ is the coordinates of the end vertex of P_i which have been iterated in previous process. $m_{\max}^i$ is the number of discrete points of the centerline of P_i. For $i = 1$, $\tilde{M}_{m_{\max}^1 \bullet}^{P_1} = M_{m_{\max}^1 \bullet}^{P_1}$. And then the $M_{j\bullet}^{P_{i+1}}$ of P_{i+1} is updated as

$$\tilde{M}_{j\bullet}^{P_{i+1}} = M_{j\bullet}^{P_{i+1}} + V_t^i, j = 1, \ldots 5m_{\max}^i \tag{23}$$

For a L_{nC} with a shape of regular polygon. The number of the edges of the polygon is $\frac{n}{2}$:

Step1: Generate standard P s with given number n and alternative handedness as:

$$P_i = f_P\left(\beta\varphi_1, \beta\left(\theta_1 + \frac{2\pi\left(1 - i + \lfloor i/2 \rfloor 2\right)}{n}\right), \beta\left(\theta_4 + \frac{2\pi\left(i - \lfloor i/2 \rfloor 2\right)}{n}\right) \middle| \Theta\right), i = 1, \ldots n \tag{24}$$

where $\beta = \left(-1\right)^{i-1}$, $\varphi_1 = -\frac{\pi}{2}$, θ_1, θ_4 and Θ satisfy the standard geometric conditions .

Step2: Rotate P_{i+1} so that its velocity vector at head vertex coincides with the one at the end vertex of P_i. The i th rotational quaternion δQ is

$$\delta Q_i = \tilde{Q}_{m_{\max}, P_i} Q_{1, P_{i+1}}^*, i = 1, \ldots n \tag{25}$$

where $Q^{*}_{1,P_{i+1}}$ is the conjugate of the quaternion which represents the rotation of the global frame on the head vertices of P_{i+1} and $\tilde{Q}_{m_{\max},P_i}$ is the quaternion which represents the rotation of the global frame on the end vertices of P_i and have been iterated in previous process. And then the $M^{P_{i+1}}$ and $Q_{\bullet,P_{i+1}}$ is updated as:

$$\begin{cases} \tilde{M}_j^{P_{i+1}} = R\left[\delta Q_i\left(1,\ M_j^{P_{i+1}}\right)\delta Q_i^{*}\right] \\ \tilde{Q}_{j,P_{i+1}} = \delta Q_i Q_{j,P_{i+1}} \end{cases} \tag{26}$$

where $R\left[\bullet\right]$ is the real(vector) part of a quaternion.

Step3: Connect the above n P_i s in turn end to end, resulting as a L_{nC}, details are same as the one of deriving L_{nO}.

For common, we define matrix $\boldsymbol{M}^L$, containing the coordinates of all the point of one L, as:

$$\boldsymbol{M}^L = \begin{bmatrix} \tilde{\boldsymbol{M}}^{P_1} \\ \tilde{\boldsymbol{M}}^{P_2} \\ \vdots \\ \tilde{\boldsymbol{M}}^{P_n} \end{bmatrix} \tag{27}$$

We define quaternion $Q_{\bullet,L}$, containing the rotations of global frames on all the points of one L, as:

$$Q_{\bullet,L} = \begin{bmatrix} Q_{\bullet,P_1} \\ Q_{\bullet,P_2} \\ \vdots \\ Q_{\bullet,P_n} \end{bmatrix} \tag{28}$$

In summary, we define L as a set whose elements are $\boldsymbol{M}^L$ and $Q_{\bullet,L}$ as:

$$L = \left\{\boldsymbol{M}^L, Q_{\bullet,L}\right\} = f_L\left(n, P\right),\ P = \left\{P_1, P_2, \ldots P_n\right\} \tag{29}$$

It is worth to note that the handedness of P_i in P is alternative in order.

4.THE WORKFLOW OF GENERATING M_0

Here, we introduce the concept of stacking points and stacking operations, and then stack four L_{3O} （ASCB-3R） and one L_{8C} (ASCB-8P) according to a given layout to form an M_0. A stacking point is the point at which the curvature sign changes on the serpentine in each ASC. If the two arcs constituting the serpentine are the same (the radius of curvature and central angle of two arcs are equal), the stacking point is the geometric center point of the serpentine line. Moreover, the stack point order is an order in which each stack point of L is generated in sequence. We denote the specified stack point in L with the stack point order. Then, we do the stacking operation which is a stacking between ASCB-nR and ASCB-mP objects by aligning their given stacking points. In details, the generation of M_0, we added a cylinder with its axis perpendicular to the two given stacking points.

Besides, the layout design is customized under a constraint condition that it must contain a polygon and no less than three open curves connected to it (a straight line is a special case of an open curve). The layout adopted in this article is the topology of tetrachiral unit. Hence, the number of ASCB-3R and ASCB-8P is 4 and 1, respectively.

For a left-handedness resp. right-handedness of M_0, the vector angle of the head and tail vector of an open curve closest to the x-axis in the first quadrant, where coordinate system established with the geometric center of the layout as the origin, is less resp. more than 45 °.

We generate a left-handedness F whose layout is the topology of tetrachiral through two following sub-steps.

Step1: Generate four L_{3O} with shape of straight line as:

$$L_{3O,i} = f_L\left(3,P\right), P = \left\{P_j, j = 1,2,3\right\}, i = 1,2,3 \tag{30}$$

where P_j are defined as the one in equation .

Step2: Generate one L_{8C} with shape of square as:

$$L_{8C} = f_L\left(8,P\right), P = \left\{P_j^{'}, j = 1,2,...8\right\} \tag{31}$$

where $P_j^{'}$ are defined as the one in equation whose $n = 8$.

Step3: Generate four cylinders as the stacking pins connecting L_{3O} and L_{8C}. The order of the stacking points of L_{3O} and L_{8C} are (1) and (1,3,5,7). The coordinates of these stacking points are:

$$\boldsymbol{S}^{L_{3O}} = \begin{bmatrix} \boldsymbol{M}_{p1}^{L_{3O}} & \boldsymbol{M}_{p2}^{L_{3O}} & 0.5T \end{bmatrix}$$

$$\boldsymbol{S}^{L_{8C}} = \begin{bmatrix} \boldsymbol{M}_{q_1 1}^{L_{8C}} & \boldsymbol{M}_{q_1 2}^{L_{8C}} & -0.5T \\ \boldsymbol{M}_{q_2 1}^{L_{8C}} & \boldsymbol{M}_{q_2 2}^{L_{8C}} & -0.5T \\ \boldsymbol{M}_{q_3 1}^{L_{8C}} & \boldsymbol{M}_{q_3 2}^{L_{8C}} & -0.5T \\ \boldsymbol{M}_{q_4 1}^{L_{8C}} & \boldsymbol{M}_{q_4 2}^{L_{8C}} & -0.5T \end{bmatrix} \tag{32}$$

where $p = \frac{1}{6}\left\lfloor m_{\max}^{L_{3O}} \right\rfloor, q_i = \frac{8+(2i-1)m_{\max}^{L_{8C}}}{128}\left\lfloor m_{\max}^{L_{8C}} \right\rfloor, i = 1,2,\ldots 4$. $m_{\max}^{L_{3O}}$ resp. $m_{\max}^{L_{8C}}$ are the total number of the points of the central line of L_{3O} resp. L_{8C}.

Before executing stacking operations, we need to generate four stacking pins. First, we draw a discrete circle at the origin of the global frame as:

$$A_s(m) = \rho_s e^{i\left(\frac{\pi}{2}+m\frac{2\pi}{n-1}\right)} \tag{33}$$

where $m \in [0, n-1]$. We compact the coordinates of this circle as a $m_{\max} \times 3$ matrix:

$$\boldsymbol{G}^{S_b} = \begin{bmatrix} \mathbf{Re}(A_s(0)) & \mathbf{Im}(A_s(0)) & 0 \\ \mathbf{Re}(A_s(1)) & \mathbf{Im}(A_s(1)) & 0 \\ \vdots & \vdots & \vdots \\ \mathbf{Re}(A_s(m_{\max})) & \mathbf{Im}(A_s(m_{\max})) & 0 \end{bmatrix} \tag{34}$$

We make this matrix as coordinates of circular points of the bottom facet of stacking pin. Next, we shift up these points with $h = 2T + \sigma$ (σ is the gap between two line objects, here we set it as 2mm) along z axis, resulting as the circular points of the top facet of stacking pin whose coordinates can be depicted as:

$$\boldsymbol{G}^{S_t} = \begin{bmatrix} \mathbf{Re}(A_s(0)) & \mathbf{Im}(A_s(0)) & h \\ \mathbf{Re}(A_s(1)) & \mathbf{Im}(A_s(1)) & h \\ \vdots & \vdots & \vdots \\ \mathbf{Re}(A_s(m_{\max})) & \mathbf{Im}(A_s(m_{\max})) & h \end{bmatrix} \tag{35}$$

Consequently, we denote the initial position of the centers of top and bottom facets of stacking pin as:

$$\boldsymbol{G}^{S_c} = \begin{bmatrix} 0 & 0 & 0 \\ 0 & 0 & h \end{bmatrix} \tag{36}$$

We concatenate the above three matrixes as:

$$\boldsymbol{M}^{S} = \begin{bmatrix} \boldsymbol{G}^{S_c} \\ \boldsymbol{G}^{S_b} \\ \boldsymbol{G}^{S_t} \end{bmatrix} \tag{37}$$

We repeat this process four times and get four matrixes of stacking pins as $\boldsymbol{M}^{S_i}$, $i=1,2,\ldots 4$. Then we translate these matrixes so that their bottom centers coincide with corresponding stacking points of L_{8C}. So the $\boldsymbol{M}^{S_i}$ is updated as

$$\tilde{\boldsymbol{M}}_{j\bullet}^{S_i}=\boldsymbol{M}_{j\bullet}^{S_i}+\boldsymbol{S}_{i\bullet}^{L_{8C}}-\boldsymbol{M}_{2\bullet}^{S_i}, i=1,\ldots 4, j=1,\ldots 2m_{\max}+2 \tag{38}$$

And also the coordinates of the stacking points of L_{8C} is updated as:

$$\tilde{\boldsymbol{S}}^{L_{8C}}=\begin{bmatrix} \boldsymbol{M}_{q_1 1}^{L_{8C}} & \boldsymbol{M}_{q_1 2}^{L_{8C}} & -0.5T+h \\ \boldsymbol{M}_{q_2 1}^{L_{8C}} & \boldsymbol{M}_{q_2 2}^{L_{8C}} & -0.5T+h \\ \boldsymbol{M}_{q_3 1}^{L_{8C}} & \boldsymbol{M}_{q_3 2}^{L_{8C}} & -0.5T+h \\ \boldsymbol{M}_{q_4 1}^{L_{8C}} & \boldsymbol{M}_{q_4 2}^{L_{8C}} & -0.5T+h \end{bmatrix} \tag{39}$$

Step4: Rotate L_{3O} s and stack them upon the top facets of the four stacking pins. The corresponding quaternion array(for left-handedness) is

$$\delta Q_i=\left(\cos(\xi),0,0,\sin(\xi)\right), \xi=-\frac{i-1}{4}\pi, i=1,\ldots 4 \tag{40}$$

And the resulting point matrix $\boldsymbol{M}^{L_{3O,i}}$ of L_{3O} are updated as:

$$\tilde{\boldsymbol{M}}_{j\bullet}^{L_{3O,i}}=R\left[\delta Q_i\left(1, \boldsymbol{M}_{j\bullet}^{L_{3O,i}}\right)\delta Q_i^*\right]+\tilde{\boldsymbol{S}}_{i\bullet}^{L_{8C}}-R\left[\delta Q_i\left(1, \boldsymbol{S}_{i\bullet}^{L_{8O}}\right)\delta Q_i^*\right] \tag{41}$$

Finally, we define the matrix $\boldsymbol{M}^{F^{3,8}}$ as:

$$\boldsymbol{M}^F=\begin{bmatrix} \tilde{\boldsymbol{M}}^{L_{3O,1}} \\ \tilde{\boldsymbol{M}}^{L_{3O,2}} \\ \tilde{\boldsymbol{M}}^{L_{3O,3}} \\ \tilde{\boldsymbol{M}}^{L_{3O,4}} \\ \boldsymbol{M}^{L_{8C}} \\ \tilde{\boldsymbol{M}}^S \end{bmatrix} \tag{42}$$

In summery , we define F as a set whose elements are $\boldsymbol{M}^F$ as:

$$F=\left\{\boldsymbol{M}^F\right\}=f_F\left(L,\zeta\right), L=\left\{L_{3O}, L_{8C}\right\} \tag{43}$$

where $\zeta=\pm 1$ for left- or right-handedness of the M_0.

5.THE WORKFLOW OF GENERATING M_{3D}

We combine multiple F s to form a polyhedron V. The choice of polyhedron determines the boundary shape of each F, and then gives the number of L_{nO} and the shape of L_{mC}. There is an adjustable space for specific parameters within the allowable range of the boundary. Moreover, the choice of polyhedron is not unique, without loss of generality, the polyhedron adopted in this article is a cube. The corresponding boundary shape of F is a square generated in above step.

To obtain the facets handedness configuration $\boldsymbol{w}$,which is the configuration of chirality arranged on each facet, which is a $1\times n$ vector, and $w_i = \pm 1$ for left- or right-handedness, we specified the serial number of each face of the V cube. We set the origin of reference coordinate system at the geometric center of the cube. Each axis of the system is perpendicular to the faces of cube. The faces whose normal vector is the same as the positive direction of the x, y, and z axes are face-1, face-2, and face-3(top face), respectively. Correspondingly, the remaining faces are face-4, face-5, and face-6(bottom face), respectively. The above facets are noted as $V^i, i = 1,2,\ldots 6$.

Each V^i has four connection points that can be connected to adjacent V^i. In order to ensure the stability of the cubic structure, each V^i needs to be connected to at least two adjacent V^i, and the connecting points are symmetrical about its geometric center, resulting in two valuable schemes $\boldsymbol{w} = \pm(-1,-1,1,-1,-1,1)$.

We generate a M_{3D} whose shape is a cube through two following sub-steps.

Step1: Generate six F^* as a face library. whose geometric center locates at the origin of the global frame.

$$F_i^* = f_F\left(L, w_i\right), L = \{L_{3O}, L_{8C}\} \tag{44}$$

Step2: Rotate and translate the above F^* s to form a cube. Here, we set the geometric center of this cube at the origin of the global frame and the normal vectors of its facets form a matrix as

$$\boldsymbol{v}^n = \begin{bmatrix} 1 & & \\ & 1 & \\ & & 1 \\ -1 & & \\ & -1 & \\ & & -1 \end{bmatrix} \tag{45}$$

The corresponding quaternion array(for left-handedness) is

$$\delta Q_i = \left(\cos\left(\frac{\phi}{2}\right), \sin\left(\frac{\phi}{2}\right)\boldsymbol{z}_i\right), i = 1,\ldots 6 \tag{46}$$

where $\phi = \cos^{-1}(\boldsymbol{e}_1 \cdot \boldsymbol{v}_{i\bullet}^n)$ and $\boldsymbol{z}_i = \boldsymbol{e}_1 \times \boldsymbol{v}_{i\bullet}^n$. First, we transfer F_i^* so that its geometric center

$$M_c^{F_i^*} = \left(\frac{\max(M_{\bullet 1}^{F_i^*}) + \min(M_{\bullet 1}^{F_i^*})}{2}, \frac{\max(M_{\bullet 2}^{F_i^*}) + \min(M_{\bullet 2}^{F_i^*})}{2}, \frac{\max(M_{\bullet 3}^{F_i^*}) + \min(M_{\bullet 3}^{F_i^*})}{2}\right) \tag{47}$$

coincidence with

$$\tilde{\boldsymbol{M}}_{j\bullet}^{F_i^*} = \boldsymbol{M}_{j\bullet}^{F_i^*} - M_c^{F_i^*} \tag{48}$$

Next, we apply affine transformation on each of these F^* as:

$$\tilde{\tilde{\boldsymbol{M}}}_{j\bullet}^{F_i^*} = R\left[\delta Q_i \left(1,\ \tilde{\boldsymbol{M}}_{j\bullet}^{F_i^*}\right) \delta Q_i^*\right] + \tau_i \boldsymbol{v}_{i\bullet}^n \tag{49}$$

where $\tau_i = \frac{1}{2}\left(\max(M_{\bullet k}^{F_i^*}) - T\right), k = 1,2,3$.

In summary, we define V as a set whose elements are $\boldsymbol{M}^V$ as:

$$\boldsymbol{M}^V = \begin{bmatrix} \tilde{\tilde{\boldsymbol{M}}}^{F_1} \\ \tilde{\tilde{\boldsymbol{M}}}^{F_2} \\ \tilde{\tilde{\boldsymbol{M}}}^{F_3} \\ \tilde{\tilde{\boldsymbol{M}}}^{F_4} \\ \tilde{\tilde{\boldsymbol{M}}}^{F_5} \\ \tilde{\tilde{\boldsymbol{M}}}^{F_6} \end{bmatrix} \tag{50}$$

6.THE WORKFLOW OF GENERATING VARIED MODULAR

To start with, we generate the varied geometry of L_{3O} undergoing simple fixed condition locating at its first stacking point and displacement condition locating at its tail vertex. So, the problem statement of L_{3O} is

$$\min_{\delta\theta_{i1},\delta\theta_{i4}} \sum_{i=1}^{3} (\delta\theta_{i1}{}^2 + \delta\theta_{i4}{}^2)$$

$$\textit{Subject to} \quad g_1\left(\delta\theta_{ij}\right) = 0$$
$$g_2\left(\delta\theta_{ij}\right) \geq 0$$

with $g_1(\delta\theta_{ij}) = e^{i(\frac{3}{4}\pi+\tau)}(\rho_{13} + \hat{\rho}_{14}e^{-i\pi} + e^{i(-\frac{1}{4}\pi-\delta\theta_{14})}(\hat{\rho}_{14} + \rho_{21}e^{-i\pi} +$
$e^{i(-\frac{1}{4}\pi-\delta\theta_{21})}(\hat{\rho}_{21} + (\rho_{22} + \rho_{23})e^{-i\pi} + \hat{\rho}_{24} + e^{i(\frac{1}{4}\pi+\delta\theta_{24})}(\hat{\rho}_{24}e^{-i\pi} + \hat{\rho}_{31} +$
$e^{i(\frac{1}{4}\pi+\delta\theta_{31})}(\hat{\rho}_{31}e^{-i\pi} + (\rho_{32} + \rho_{33}) + e^{i(-\frac{5}{4}\pi-\delta\theta_{34})}\hat{\rho}_{34}))))) - U$

$g_2(\delta\theta_{ij}) = \delta\theta_{i1}\delta\theta_{i4} \geq 0, i = 2,3$

where $\tau = \delta\theta_{14} + \delta\theta_{21} - \delta\theta_{24} - \delta\theta_{31} + \delta\theta_{34}$ .The auxiliary conditions are

(i) $\delta\theta_{11} = 0$;

(ii) $\hat{\rho}_{ij} = \rho_{ij}(\theta_{ij} + \delta\theta_{ij}) / \theta_{ij}, i = 1,2,3, j = 1,4$ where ρ_{ij}, θ_{ij} is the radius and circular angle of the ASC of the jth arc of the ith P in L_{3O} under standard conditions, respectively;

(iii) $U = U_x + iU_y$, the given displacement condition constraining on the end vertex of L_{3O};

(iv) The standard condition aforementioned in above section.

Next, we generate the varied geometry of M_0 undergoing four displacement conditions locating at its four-tail vertex. The magnitude of the displacement conditions are equal. We divided M_0 into four parts, each of them is consisted of one corner of L_{8C} containing two P's and one L_{3O} containing three P s. The problem statement of F^* is

$$\min_{\delta\theta_{i1},\delta\theta_{i4}} \sum_{i=1}^{8} (\delta\theta_{i1}{}^2 + \delta\theta_{i4}{}^2)$$

$$\textit{Subject to} \quad g_1\left(\delta\theta_{ij}\right) = 0$$
$$g_2\left(\delta\theta_{ij}\right) \geq 0$$

with $$g_1(\delta\theta_{ij}) = -\frac{1}{2}e^{i(\frac{3}{4}\pi+\tau)}(\rho_{13} + \hat{\rho}_{14}e^{-i\pi} + e^{i(-\frac{1}{2}\pi-\delta\theta_{14})}(\hat{\rho}_{14} + \rho_{21}e^{-i\pi} +$$
$$e^{i(-\frac{1}{2}\pi-\delta\theta_{21})}(\hat{\rho}_{21} + (\rho_{22} + \rho_{23})e^{-i\pi} + \hat{\rho}_{24} + e^{i(\frac{1}{4}\pi+\delta\theta_{24})}(\hat{\rho}_{24}e^{-i\pi} + \hat{\rho}_{31} +$$
$$e^{i(\frac{1}{4}\pi+\delta\theta_{31})}(\hat{\rho}_{31}e^{-i\pi} + (\rho_{32} + \rho_{33}) + \hat{\rho}_{34}e^{i(-\pi)} + e^{i(-\frac{1}{2}\pi-\delta\theta_{34})}(\hat{\rho}_{34} + \hat{\rho}_{41}e^{i(-\pi)} +$$
$$e^{i(-\frac{1}{2}\pi-\delta\theta_{41})}(\hat{\rho}_{41} + (\rho_{42} + \rho_{43})e^{i(-\pi)} + \hat{\rho}_{44} + e^{i(\frac{1}{4}\pi+\delta\theta_{44})}(\hat{\rho}_{44}e^{i(-\pi)} + \hat{\rho}_{51} +$$
$$e^{i(\frac{1}{4}\pi+\delta\theta_{51})}(\hat{\rho}_{51}e^{i(-\pi)} + \hat{\rho}_{52})))))))) + e^{i(\frac{3}{4}\pi+\tau)}(\rho_{63} + \hat{\rho}_{64}e^{-i\pi} + e^{i(-\frac{1}{4}\pi-\delta\theta_{64})}(\hat{\rho}_{64} + \rho_{71}e^{-i\pi} +$$
$$e^{i(-\frac{1}{4}\pi-\delta\theta_{71})}(\hat{\rho}_{71} + (\rho_{72} + \rho_{73})e^{-i\pi} + \hat{\rho}_{74} + e^{i(\frac{1}{4}\pi+\delta\theta_{74})}(\hat{\rho}_{74}e^{-i\pi} + \hat{\rho}_{81} +$$
$$e^{i(\frac{1}{4}\pi+\delta\theta_{81})}(\hat{\rho}_{81}e^{-i\pi} + (\rho_{82} + \rho_{83}) + e^{i(-\frac{5}{4}\pi-\delta\theta_{84})}\hat{\rho}_{84}))))) - U$$

$$g_2(\delta\theta_{ij}) = \delta\theta_{14}\delta\theta_{21} \geq 0, i = 2,3$$

where $\tau = \delta\theta_{64} + \delta\theta_{71} - \delta\theta_{74} - \delta\theta_{81} + \delta\theta_{84}$ .The subscript $i = 1,2,...5$ of $\delta\theta_{ij}$ represent the ASCs of the five P' s belonging to L_{8C} and $i = 6,7,8$ represent the ASCs of the three P s belonging to L_{3O} .The auxiliary conditions are

(i) $\delta\theta_{11} = \delta\theta_{61} = 0$

(ii) $\hat{\rho}_{ij} = \rho_{ij}(\theta_{ij} + \delta\theta_{ij}) / \theta_{ij}, i = 1,2,...8, j = 1,4$ where ρ_{ij}, θ_{ij} is the radius and circular angle of the j th arc of the i th ASC in L_{8C} and L_{3O} under standard conditions, respectively. The subscript $i = 1,2,...5$ of ρ_{ij} and θ_{ij} represent the ASCs of the five P' s belonging to L_{8C} and $i = 6,7,8$ represent the ASCs of the three P s belonging to L_{3O}.

(iii) $U = U_x + iU_y$, the given displacement constraints on the end vertices of F^*.

(iv) The standard conditions aforementioned in above section.

Finally, we generate the varied geometry of V^* undergoing volumetric expansion/shrinking. As we have mentioned in main text, we fixed the shape of all six L_{8C} s and adjust the shape of the rest L_{3O} s to realize this variational design. We transfer the displacement of the tail vertex U of the L_{3O} of the F^* to the angle of the rotation of the L_{8C} of the F^* which can be simplified one input variable to control the whole shape of V^*. We implemented this idea as following transferring:

$$U = \sqrt{D_x^2 + D_y^2} e^{i \tan^{-1}\left(\frac{D_x}{D_y}\right)} \left(e^{i\psi} - 1\right) \quad (51)$$

where $D = D_x + D_y$ is the position of the tail vertex of L_{3O} in the F^* under standard conditions. We implemented a harmonic search algorithm by MATLAB and solve the above problem statements.

7. Supplementary Figures

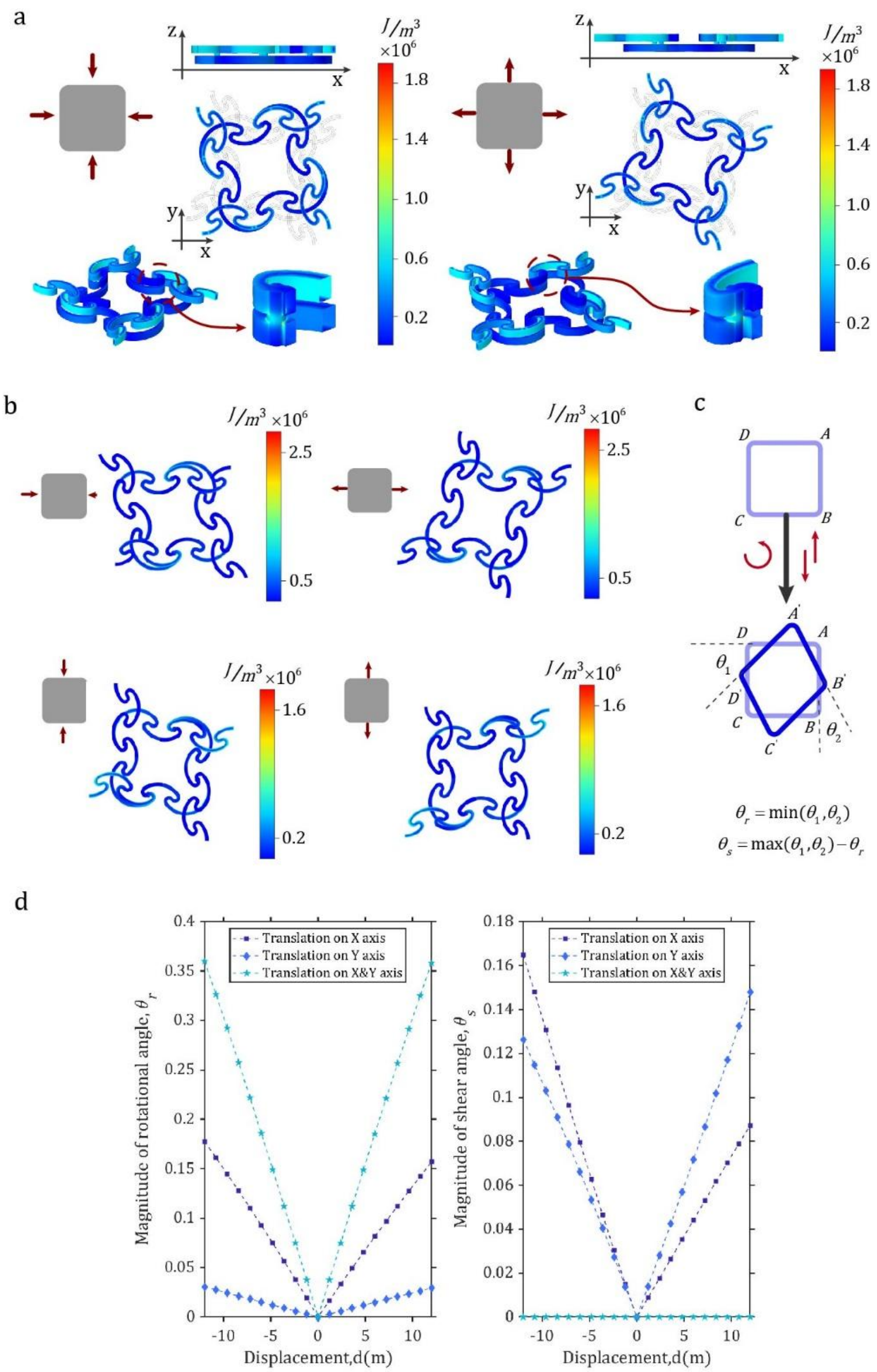

Figure S1. The simulation results of M_0 undergoing two types of boundary conditions. (a) The top-, front- and perspective view of the deformation of M_0 that undergoes compression and stretching along the X- and Y-axis simultaneously. (b) The top-, front- and perspective view of the deformation of M_0 that undergoes compression and stretching along the X-axis and Y-axis, respectively. (c) The definition of rotational angle and shear angle of M_0. (d) The angle-displacement data of M_0 undergoing above boundary conditions. In all six scenes: The colored counter shows the distribution of the elastic energy density caused by deformation. We applied prescribed displacements on the four or two vertexes of the face object and maintained the rest free.

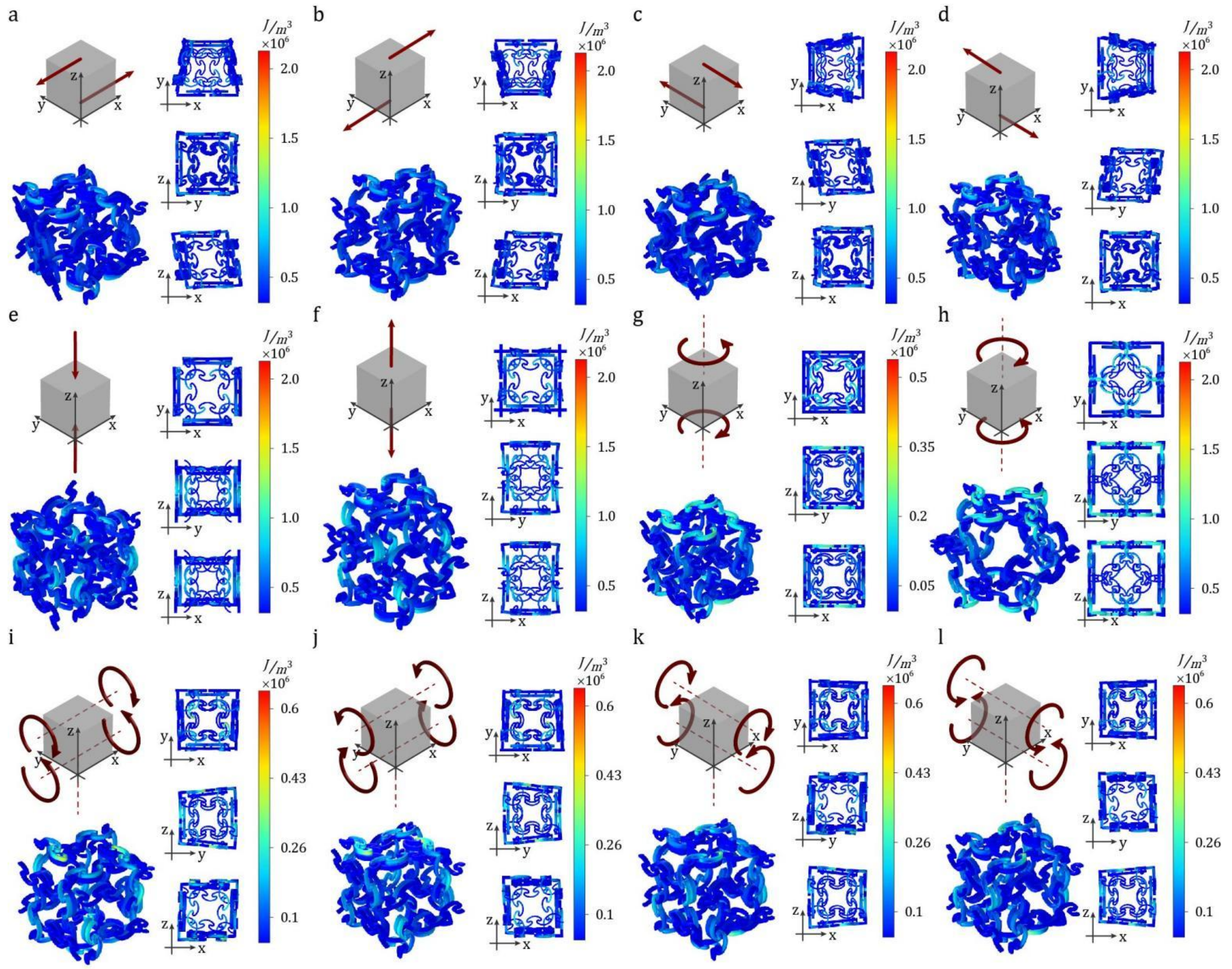

Figure S2. The simulation results of M_{3D}-I whose acted facets are top face and bottom face. (a-f) The top-, front-, left-, and perspective view of the deformation of M_{3D}-I undergoing linear prescribed displacements along the X-, Y-, and Z-axis, respectively. (g-l) The top-, front-, left-, and perspective view of the deformation of M_{3D}-I undergoing prescribed rotations along the X-, Y-, and Z-axis, respectively. In all scenes: The colored counter shows the distribution of the elastic energy density caused by deformation. We applied prescribed displacements on top face and bottom face of M_{3D}-I and maintained the rest free.
